# The Essential Oils, and Eucalyptus Globulus in Dentistry

**Published:** 1882-04

**Authors:** A. W. Harlan

**Affiliations:** Chicago, Ill.


### THE ESSENTIAL OILS; AND EUCALYPTUS GLOBULUS IN DENTISTRY.

BY A. W. HARLAN, D.D.S.
Chicago, Ill.

In venturing to call attention to the virtues of the within mentioned substances it may be thought that most of them have been well investigated, but a new examination may be not uninteresting. The history of the essential oils here dwelt upon, is explicit in saying that they have already been more or less used in dental surgery, and all that I shall say of them is, that having tried all of them for purposes to be hereafter mentioned, I am able to supplement what the books say relative to their merits.

The volatile oils usually emit an odor resembling that of the plants from which they have been distilled, possessing no chemical or physical property common to the fixed oils; but, like the latter, they are generally soluble in ether, chloroform, bisulphide of carbon, etc., having a specific gravity less than water, with one or two exceptions. They are all inflammable and should be protected from light and air; the addition of about three per cent. of alcohol prevents oxidation. The composition of these oils is usually represented by CH or CHO. Those composed of $C_{10}H_{14}$ or multiples thereof, are called terpens, and the oxigenated oils are those from whence may be seperated a dark-blue body called azuline, the composition of which is $C_{16}H_{26}O$, or a brownish or yellowish resin, which gives to the oil its characteristic color. These oils, when dropped upon paper, leave a stain which disappears when the paper is heated; the fixed oils permanently stain the paper. A test for adulteration is to dissolve the oil in alcohol, if there be any residue it is impure or a fixed oil; if the oil is adulterated with alcohol, dry acetate of potassium will dissolve in the suspected oil, or fused calcium chloride, neither of which is soluble in a volatile oil. There are other methods of detecting adulterations, but a better way is to purchase from a responsible druggist.

The oils of peppermint and cloves have long been used by the public and by dentists, the former using them to stop toothache and the latter for the same purpose at times, but oftener as a dressing for cavities in teeth, not as pain obtunders, *per se*, but for the purpose of arresting pain. Clove oil is the favorite with the public and also with a large number of dentists, but it is not of either of those oils that I desire to speak, but of the oils of cajeput, savin, pimento, anise, horsemint, origanum, caraway and the oil of eucalyptus globulus. The first seven out of a long list of volatile oils are those possessing in the greatest degree the local anæsthetic principle. Now, in order to make the best use of any remedial agent or pain obtunder it is necessary to exclude moisture and dry the cavity, but in the limited use I have made of these oils (six months) I much prefer cajeput or origanum over others, even though the cavity be not wholly dry, neither of them being escharotic or nauseous to the most delicate. They will arrest the pain in a tooth caused by change of temperature, sweet, sour or external cause, almost instantly, and the effect is more lasting than that from chloroform or any of the liniment dressings which I have used in similar cases, not excepting the hydrate of chloral and camphor mixture, each ℥ *ss.* forming a liquid. As cajeput is likely to be much used in dentistry a brief history of it is here presented:

The oil is distilled from the leaves of a small tree indigenous in the East Indies, the Phillippines and portions of Australia; it is a limpid greenish colored oil with a penetrating odor, resembling the combined odors of camphor, rosemary and mint, with a cool camphorous taste; it is very soluble in alcohol, and does not affect litmus; it dissolves iodine, red rubber and pure gutta-percha, but not gutta-percha used for filling. Its composition is $C_{10} H_{16} H_2 O$ and boils at 344 far.; it is subject to adulteration by turpentine, which hinders its dissolution in alcohol, it is not very expensive, and there are now used in the United States about

1,500 pounds annually. It is used in medicine as a stimulant and diaphoretic, for flatulent colic, chronic rheumatism, paralysis, etc. It is used locally diluted with olive oil or glycerine in functional paralysis and muscular rheumatism. Dentists use it for the relief of otalgia and deafness by introducing it into the auditory canal. A few drops rubbed on the temples relieves a nervous headache, or by rubbing it over the supra-orbital nerves at their exit it arrests the pain of facial neuralgia. I know it to be a pain obtunder when there is a pulp exposure, or if a tooth be aching without an exposure it instantly relieves it. I am unable to say as yet how good an obtunder of sensitive dentine it is, but in a few cases it has been found very satisfactory, especially in the teeth of young persons. Patients do not object to the odor, as it is new to most of them and very persistent; it is mildly antiseptic and merits a trial. The oils of origanum and caraway I have used experimentally as pain obtunders, and in several cases persons have stated that the pain was much abated by their use, but not wholly subdued. Oil of origanum is very nearly allied to the oil of thyme, but is not as good an antiseptic. It is stimulant and carminative, and has for composition $(C_{10} H_{16})_5 O$. In one case I was enabled to cut thoroughly and freely where it was very difficut to cut at all before using it. It is cheap. The oil of caraway is the substance from which carvicrol is obtained, and is poisonous in large doses, one and one-half ounce causing the death of a mastiff by asthma. It is agreeable to the taste, and is very anæsthetic when applied to the mucus membrane. A combination of caraway and cajeput, each ℥ss, makes a very good obtunder. I have used it in ten cases and am well pleased with it. Its application to a dry cavity is a necessity to render it soluble.

Oil of anise, as is well known, is used very largely in medicine for chronic bronchitis—to prevent griping after the use of purgatives, and to relieve colic and flatulence. About 2,400 lbs. are imported annually. It has not been used in dentistry, but equal parts of anise, caraway and oil of fennel, I am persuaded, are about the proportion in which anise may become useful as a pain obtunder. By itself it relieves toothache, and the odor of the oil remains for twenty-four hours in the presence of a small quantity of sulphuretted hydrogen. It makes an agreeable dressing compound with oil of cloves. Oil of savin is poisonous in quarter ounce doses, when given to animals, and should be used with caution. It is astringent and anæsthetic. Applied to an exposed pulp, it forms a protective covering, which is not insoluble as the oil is not escharotic. It is a good pain obtunder, and may be rubbed over the temples, combined with the oil of caraway, peppermint, or anise, to assuage the pain in facial neuralgia. I have used it with good effect in six cases for excavating sensitive dentive; it is a powerful stimulant, and when twenty drops are added to thirty of iodine and five of carbolic acid it may be used with gratifying results applied to tumefied gums and mouth lesions from stomachic derangement. Oil of pimento is highly aromatic and stimulant, and may be used for infecting abscesses connected with deciduous teeth; it is more agreeable then cloves and is recommended for use in a mouth wash for aphtheous ulcers combined as follows;

| | | |
|---|---|---|
| Oil Pimento.......................... | Minims | X |
| Carbolic acid.......................... | " | V |
| Caustic Soda.......................... | Grs. | iij |
| Water add qs.......................... | | iij |

Dilute to tast.

It ranks with oil of cloves as a pain obtunder. Oleum monardea or horsemint is an excellent pain obtunder, but it also is poisonous; the dose internally being three drops, in sweetened water. It should be applied when the rubber dam is over the teeth. I like it very much, having used it sparingly at intervals during six months Its composition is the same as that of oil of thyme; the smallest drop applied to the tongue "diffuses over it and the faucies a pungent aromatic and persistent heat." Vesication may be produced with it and a saturated solution in iodine can be used to paint the gums over a tooth affected by pericementitis as a counter irritant. It is a good local remedy for facial neuralgia and is more permanent in its effects than any of the volatile oils. The oil of eucalyptus globulus comes from the blue gum tree of Yosmonia, and from Victoria, on the mainland of Australia. It has been cultivated in the U. S. in the South and Southern California; the tree attains a height of 300 feet. This remedy is purchasable in three forms, the extract, a dark colored liquid with a smell like opium, the oil and a volatile extract. I have used these three forms of the substance and am prepared to say that from my use of them the volatile extract is the form, which is to be prefered for local use. After opening a pulp cavity from whence no discharge issues, but a foul odor is emitted, a dressing of cotton saturated with the extract and protected by wax, sandarach or gutta-percha; after two or three days has elapsed, reveals the fact, that the odor from the tooth has disappeared and the odor of the extract remains. Now, I have used it side by side with carbolic acid, solution of cloride of zinc, iodide of zinc and glycerole of thymol in the treatment of blind abscess and the treatment of pulpless teeth without abscess and I believe it to be equally as useful as the best of the foregoing remedies. As an injection into a blind abscess it acts most admirably, it is more penetrating than iodine and its odor is so persistent, that weeks and months afterwards you may detect it, when only protected by sandarach on cotton it does not stain the dentine and is not escharotic, two very good qualities in a remedy proposed for the treatment of non-fistulous abscesses. Applied to spongy gums every other day they soon became reduced in size; but it is as a deodorizer, that I am particularly pleased with it. A drop or two in a quarter tumbler of water may be used to rinse the mouth, when the breath is foul. If it is inhaled for five minutes three or four times a day it benefits those suffering from nasal catarrh; in the necrosis of the process and ulcers of the mouth it ranks by the side of the oil of thyme and the Listorian carbolic dressing. Those who may not have used it will be well repaid by using it to treat cases of blind abscess where great care is necessary, to prevent suppuration. As it dissolves red or black rubber, other methods must be resorted to for injecting it into abscesses. When it is not practicable to inject with a syringe or by using a broach armed with cotton to pump it through the canal, a piece of spunk placed over the moistened pellet of cotton is covered by a piece of linen tape and then by using soft rubber to fill the whole of the cavity, pressure on that forces the remedy through the canal into the sac and prevents staining the tooth with dissolved rubber. In the treatment of such cases care must be used to prevent saliva or water from gaining access to the canals, the teeth should always be kept dry by using the rubber dam and the canals washed with alcohol and dried before applying the extract.

### FILLING TEETH WITH COHESIVE FOIL.

BY E. G. BETTY, D.D.S.

(Read before the Mississippi Valley Dental Society, March 2, 1882.)

Mr. President:—Your Executive Committee has seen fit to honor me with an appointment among the essayists upon the programme, and while I deem it the duty of every member to contribute towards the general fund, I am, nevertheless, of the opinion that an older and more experienced head should have been selected to execute the task relegated to me.

Besides this, the subject-matter is one that few are now fain to write about; not that it is difficult at all, but from the fact that it has been so belabored and rehashed that the average dentist turns up his nose in disgust, when the very name is mentioned.

It is, however, a subject of more practical importance to the dentist than any other usually discussed at meetings such as this, and one that is more talked about, and least understood. From the very nature of things, it becomes almost impossible to treat of it, without the remarks degenerating into a mere description of individual methods at the chair. Each and every one of us is apt to consider his own peculiar method of operating as the *ne plus ultra* of professional attainment, and cannot understand why it is that his ideas are not at once adopted by the profession at large. The attempt, then, upon the part of one whose experience is of "tender years," to correct what he deems hurtful to the efficiency of an operator, would seem to imply an egotism that does not exist.

Were all writers and speakers to cease advancing their ideas, for fear of incurring the charge of egotism, an Egyptian darkness would soon obscure all that is bright, progressive and useful.

Thus it is that a hesitation is felt in approaching a subject that contains nothing in the nature of news, and a great deal upon which a reputation is either made or wrecked.

So diverse and deeply rooted are opinions and methods of procedure that one can scarcely hope to bring out of the existing chaos anything bearing the semblance of uniformity, in the handling of material for filling.

The several men of note in the profession, whose modes of operating might be mentioned in illustration of this point, all have more or less experience in their particular lines, and are looked upon by their followers as standard authority. They expound their methods in the journals and at society meetings, and are applauded just in proportion as they harmonize with the views of their auditors. Each advocates some one or other specific method of manipulation, or brand of material to use, and are the means of doing much good, or incalculable harm, when they are measured by the results that obtain.

It is not the present purpose to enter into detailed specifications as to how a filling should be built, nor to advertise in an indirect way any particular make of foil.

We all have a more or less correct idea as to what is necessary to do in the operation we call filling. With the *idea*, however, we have less to do than with the *manner* in which the work is done, and the form of material with which it is accomplished.

The slouch leaves his disgraceful mark upon every tooth he touches, and deludes not only his patients, but himself as well, that the work is done in a masterly fashion. He prates loudly in the meetings, talks with grace and apparent candor; is at great pains to heartily coincide with any really good operator who happens to describe a fine operation, and remarks "that is just the way I do." Possibly, but when you come to analyze him by the evidence of his handiwork, the proof is plain that his conception of what he has heard is limited by his meager powers of execution. For this reason it avails but little how much stress is laid upon the importance of this, that, or the other step in an operation.

There is a kind of conservative, who is frequently "too utterly utter," and manages to preserve so much of a decayed tooth that there is no room for the introduction of a filling worthy of the name. In fact, he is guided by the direction the decay has taken, and has not the courage to deviate from that course. The cavity and its walls are prepared by the decay, and with such a preparation for filling, we necessarily are not surprised to see it stuffed with cylinders of soft foil, or a bolus of seductive crystal gold. Such plugs, of course, are not calculated either to save the tooth or subserve the purposes of the destroyed portions. They can have no integrity in themselves, and certainly do not obtain any by catalytic action from the teeth containing them. Undoubtedly, a decayed tooth, if left alone, will surely be annihilated. What folly, then, to tinker and fiddle around, trying to save every crumb of tooth-structure that does not happen to be actually saturated with rottenness.

The tooth is sick,--sick unto death; part of its structure is obliterated, and if radical measures are not at once adopted, there remains no hope for its salvation. Chisel and file should be unsparingly used until every advantage possible is gained, by putting into proper shape the remainder of the tooth. None but the timorous hesitate to cut away sound tooth-substance, when by so doing direct access and vision into the cavity may be had, and every advantage secured to make the filling as it *ought* to be made. *Everything in the way of excavation that will add to the ease and thoroughness of the introduction of the gold, will proportionately increase the preserving power of the plug.* The operator who is conscious of an ability to restore lost parts with foil, will see the necessity of a radical excavation of the tooth he is about to redeem. The more solid parts of the tooth are the ones upon or in which to make anchorages. If one or two strong parts remain, they will ordinarily be sufficient foundation upon which to restore lost parts, and also obtain protection for themselves.

The details of grooves, dovetails, angles and retaining-pits, have been reiterated so frequently that mere mention of them will suffice.

There remains, now, to consider the form of gold to be used. If but two walls are standing, or the subject for restoration is a mere root, soft foil, in any shape, is out of the question. Our hope, then, rests upon what is known as cohesive foil. And right here is one of the most stubbornly contested points we are expected to pass upon. The number of brands is legion, further multiplied by the number of weights made, and the infinite variety of forms in which the foil is prepared for use. All forms of blocks, cylinders, pellets, ropes and ribbons, are simply foil with another name. To make a selection, then, of the form of foil that will secure the best results, involves a study of the requirements that are to be met. The foil, when introduced, should maintain a permanent integrity in spite of all causes militating against it. This permanent integrity includes, of course, indestructibility, hardness, tenacity and cohesion, for if one or other of these elements were abstracted no plug would remain intact.

All forms of foil possess some one or more of these qualities, but they greatly differ in the degree, and it is for this reason that a discriminating intelligence must be exercised in determining what form possesses all these attributes in the highest perfection.

The metal, gold, has certain properties that never desert it, no matter what shape it is worked into; as, for instance, color, specific gravity, thermal and electric conductivity, etc. With these, however, we have nothing to do, as they are ever present, and are conditions and susceptibilities beyond our control. It is the more mechanical properties that concern us most, they being the ones we can modify to some extent to suit our wishes.

The molecular force, cohesion, exerts a greater influence over the results of our operations than any other property of gold, and is *the* one upon which the whole system of filling depends, having, in fact, only a few years ago, caused a complete revolution in applied dental science. If studied closely, it is not difficult to trace to cohesion the hardness, tenacity, ductility, malleability, etc., of the gold as a metal, and when in the form of a plug its integrity, indestructibility and preserving power. This, then, is the prime factor that must determine us in the selection of the form and weight of foil with which to construct our fillings.

Granting the premise, it may be laid down as a principle that *the fewer the surfaces brought together the greater will be the resisting*

*power of the mass.* Experience has proved this to be a fact in many departments of industrial pursuits, and why not in dentistry as well?

With this principle in view, we may at once dismiss all forms of crystal gold, their granular nature multiplying indefinitely the number of surfaces to be united. At all events, that form of gold cannot be included under the term foil.

To put our principle into practice, implies an increase in the thickness, and, consequently, of the weight of the foil that is to be used. It may be charged in this connection that, assuming the necessity of fewer surfaces to weld, the foil may be augmented in weight and thickness to an unlimited degree.

Very true; but it must not be forgotten that beyond a certain point we would lose a very important property, indispensable to the preserving power of the foil; namely, adaptability. We must, then, adopt a medium weight, one that will combine the advantage of a reduced number of surfaces with an easy adaptability. These are best had, in all probability, in that foil called number sixty. This weight, in the estimation of a great many operators, possesses in the most eminent degree a combination of all the qualities necessary for a perfect filling. A single strip of it is equal in weight to a pellet or block made of number two foil folded thirty times, or a block of number four folded fifteen times.

The block of number two has sixty surfaces to be welded; that of number four has thirty. Each layer of this light foil is weak just in proportion to its tenuity, its little strength still further reduced by the strain necessary to keep in contact its sixty or thirty surfaces. How much further the cohesion of a plug made of this foil is reduced when the contact surfaces are multiplied an hundred fold!

The same is true of plugs made of ribbons or ropes of light foil. No wonder, then, that fillings made of light numbers crumble under the plugger and disappear when subjected to the craunching process of mastication. As a means of restoring lost structure, especially contouring, they are of little value, soon losing their cohesion and disintegrating in consequence. In bold contrast with them stands the reliable heavy-weight. It has but two surfaces to weld together; its bulk is reduced to a fraction; it will stand as much malleting as the tooth, without losing its receiving surface; it is hard and tough to the last degree, and will wear for years, all the while maintaining its solidity and outline uninjured.

Believing that more good is accomplished by a radical excavation of cavities and the use of a foil that *is* foil, the hope is expressed that it will come into general use. We will then hear less complaint about imperfect cervical joints, broken corners, and the whole list of calamities that usually attend the performances with æsthetic forms of gold.

